# *Mtb*-Specific CD27^low^ CD4 T Cells as Markers of Lung Tissue Destruction during Pulmonary Tuberculosis in Humans

**DOI:** 10.1371/journal.pone.0043733

**Published:** 2012-08-24

**Authors:** Irina Yu Nikitina, Natalya A. Kondratuk, George A. Kosmiadi, Rasul B. Amansahedov, Irina A. Vasilyeva, Vitaly V. Ganusov, Irina V. Lyadova

**Affiliations:** 1 Department of Immunology, Central Tuberculosis Research Institute, Moscow, Russia; 2 Department of Radiology, Central Tuberculosis Research Institute, Moscow, Russia; 3 Department of Phthisiology, Central Tuberculosis Research Institute, Moscow, Russia; 4 Department of Microbiology, University of Tennessee, Knoxville, Tennessee, United States of America; 5 National Institute for Mathematical and Biological Synthesis (NIMBioS), Knoxville, Tennessee, United States of America; University of Cape Town, South Africa

## Abstract

**Background:**

Effector CD4 T cells represent a key component of the host’s anti-tuberculosis immune defense. Successful differentiation and functioning of effector lymphocytes protects the host against severe *M. tuberculosis* (*Mtb*) infection. On the other hand, effector T cell differentiation depends on disease severity/activity, as T cell responses are driven by antigenic and inflammatory stimuli released during infection. Thus, tuberculosis (TB) progression and the degree of effector CD4 T cell differentiation are interrelated, but the relationships are complex and not well understood. We have analyzed an association between the degree of *Mtb*-specific CD4 T cell differentiation and severity/activity of pulmonary TB infection.

**Methodology/Principal Findings:**

The degree of CD4 T cell differentiation was assessed by measuring the percentages of highly differentiated CD27^low^ cells within a population of *Mtb*- specific CD4 T lymphocytes (“CD27^low^IFN-γ^+^” cells). The percentages of CD27^low^IFN-γ+ cells were low in healthy donors (median, 33.1%) and TB contacts (21.8%) but increased in TB patients (47.3%, p<0.0005). Within the group of patients, the percentages of CD27^low^IFN-γ^+^ cells were uniformly high in the lungs (>76%), but varied in blood (12–92%). The major correlate for the accumulation of CD27^low^IFN-γ^+^ cells in blood was lung destruction (r = 0.65, p = 2.7×10^−7)^. A cutoff of 47% of CD27^low^IFN-γ^+^ cells discriminated patients with high and low degree of lung destruction (sensitivity 89%, specificity 74%); a decline in CD27^low^IFN-γ^+^cells following TB therapy correlated with repair and/or reduction of lung destruction (p<0.01).

**Conclusions:**

Highly differentiated CD27^low^ Mtb-specific (CD27^low^IFN-γ^+^) CD4 T cells accumulate in the lungs and circulate in the blood of patients with active pulmonary TB. Accumulation of CD27^low^IFN-γ^+^ cells in the blood is associated with lung destruction. The findings indicate that there is no deficiency in CD4 T cell differentiation during TB; evaluation of CD27^low^IFN-γ^+^ cells provides a valuable means to assess TB activity, lung destruction, and tissue repair following TB therapy.

## Introduction

Tuberculosis (TB) is one of the most common infectious diseases worldwide. Prevention of TB dissemination in the community critically depends upon efficient TB diagnostics and treatment. Current strategies to diagnose TB disease and monitor TB treatment are based on microbiological, clinical and radiographic examinations, all having limitations. Microbiological diagnosis is limited because patients with less-extensive pulmonary TB often lack identifiable *Mtb* in their sputum, or sputum is unavailable for microbiological analysis; clinical symptoms are often non-specific; chest radiography does not allow to distinguish between active TB, inactive infection, and other lung pathologies. Thus, new TB tests are needed. Ideally, such tests should be based on blood sample analysis and should evaluate TB activity, i.e., they should distinguish TB disease from latent infection and assess disease activity in patients with diagnosed TB [1]–[3].

Immunological tests based on T cell analysis have a high potential for TB diagnostics and monitoring. Immunological assays based on the evaluation of T-cell mediated IFN-γ responses (i.e., T-SPOT.TB, QuantiFERON-TB Gold) have proved to be useful for detecting *Mtb* infection [4]–[8]. Unfortunately, they have demonstrated poor ability to distinguish between active and latent TB [9]–[13], two forms of *Mtb* infection that differ in their contagiousness and treatment strategies. To overcome these limitations, new approaches have been suggested, including phenotypic analysis of IFN-γ producing CD4 T cells [14], [15] and quantification of polyfunctional and TNF-α producing CD4 T cells [16], [17]. The applicability of these assays for discriminating between active and latent infections is being tested. In contrast, tests to evaluate disease activity in patients with diagnosed TB are unavailable. This is in spite of the fact that TB may have a spectrum of activities characterized by different degrees of lung pathology and disease severity and may require alternative treatment strategies.

Our previous studies performed in a mouse model of *Mtb* infection, suggested that it is possible to evaluate the infectious process ongoing in the lungs during TB by analyzing the proportion of the CD27^low^ effector CD4 T cell subset. CD27, a member of the TNF-receptor superfamily [18], is constitutively expressed by naive T cells and early effector lymphocytes, but is downregulated at late stages of effector cell differentiation; accordingly, late effector lymphocytes exhibit low to no CD27 expression [19]–[24]. Late CD27^low^ effector T cells differentiate from CD27^hi^ effector precursors under antigenic and/or inflammatory stimuli [19], [21]. Our studies in mice have demonstrated that during *Mtb* infection, CD27^low^ effector CD4 T cells differentiate from CD27^hi^ effector precursors directly in the lungs and their differentiation is promoted by lung *Mtb* infection [24], [25]. We also showed that in mice, active *Mtb* infection leads to the accumulation of CD27^low^ effector CD4 T lymphocytes in the lungs, blood, and other organs of infected mice [24], [25]. In humans, patients with active pulmonary TB have higher frequency of CD27^low^ *Mtb*-specific CD4 T cells than latently- infected individuals [14]. Based on these observations, we hypothesized that the degree of CD27^hi^→CD27^low^ differentiation may serve as an indicator of disease activity within the lungs during TB. Here we addressed our hypothesis by evaluating CD27^low^ *Mtb*-specific CD4 T cells in patients with pulmonary TB. We show an association between *Mtb*-induced lung tissue destruction and the accumulation of CD27^low^ *Mtb*-specific CD4 T cells in the blood of TB patients and present evidence that evaluation of CD27^low^ cells provides a means to assess lung destruction and tissue repair following TB therapy.

## Methods

### TB Patients

All studies were approved by the IRB #1 of the Central Tuberculosis Research Institute of Russian Academy of Medical Sciences and were conducted in accordance to the principles expressed in the Helsinki Declaration. Seventy two patients under treatment in the CTRI gave written informed consent and were enrolled in the study. Two TB suspects with unconfirmed diagnosis were further excluded from the study. In 70 patients with diagnosed TB, diagnosis was based on clinical and/or radiographic evidence of TB plus either identification of *Mtb* in the sputum (60 patients) or response to anti-TB therapy (10 patients). Fifty one patients had recently diagnosed TB; 19 patients had chronic (>1 year) TB and had received several courses of therapy.

Of 70 patients included in the study, blood cell analysis was performed in 62 patients; in 8 patients, lung and blood cells were analyzed. Of 62 patients, 50 patients formed the main group (median age, 31 years; range 18–71 years; 30 women), and 12 patients – the validation group (median age, 31 years; range 18–76 years; 5 women, Table 1). In all 62 patients, immunological analysis was performed within 2.0±0.1 weeks of patients’ admittance to the hospital, and the clinical follow-up for these patients was at least 2 months. In 22 patients of the main and validation groups, additional immunological analysis was performed after 2-mo TB therapy (“dynamic” group). The clinical follow-up for these patients was 4 months.

**Table 1:** Characterization of groups included in the analysis.

Characteristic		TB patients^1^			*Mtb*-unexposed^1^	TB contacts^1^	
		Main groupn = 50	Validation groupn = 12	Surgerygroupn = 8	n = 15	QFT^−^n = 11	QFT^+^ n = 10
**Gender**	Female	30 (60%)	5 (42%)	4 (50%)	8 (53%)	8 (73%)	8 (80%)
**Age**	Median	31	31	34	28	41	50
	Range	18–71	18–76	21–51	19–71	25–73	28–74
**TB duration**	Recent	39 (78%)	10 (83%)	2 (25%)	NA^2^	NA	NA
	Chronic	11 (22%)	2 (17%)	6 (75%)	NA	NA	NA
***Mtb*** ** in the sputum**	*Mtb+*	43 (86%)	12 (100%)	5 (63%)	NA	NA	NA
	*Mtb−*	7 (14%)	0	3 (37%)	NA	NA	NA
**Form of TB pathology**	Tuberculoma	5 (10%)	1 (8%)	2 (25%)	NA	NA	NA
	TB infiltrate	30 (60%)	9 (76%)	0	NA	NA	NA
	Cavitary TB	10 (20%)	1 (8%)	6 (75%)	NA	NA	NA
	Caseous pn.	5 (10%)	1 (8%)	0	NA	NA	NA

1 Indicated are numbers (%).

2 NA, not applicable.

Immunological analysis of lung cells was performed in 8 patients who underwent lung tissue surgery because of extensive lung disease or to distinguish between tumor and tuberculosis lesion (“surgery” group; median age, 34 years; range, 21–51 years; 4 women). In these patients, lung derived cells, blood cells and (where available) pulmonary lymph node cells were analyzed on the day of surgery.

All enrolled patients were HIV seronegative. All analyses were performed during years 2008–2011. Detailed clinical characteristics of enrolled patients are presented in Table S1.

### TB Contacts and Healthy Donors

Healthy CTRI employees (n = 21) working in tight contacts with TB patients for at least 1 year (median, 7 years; range 1–51 years) and having no clinical or radiographic evidence of tuberculosis were enrolled in the study as *Mtb*-exposed individuals (“TB contacts”; median age, 43 years; range 25–74 years; 16 women; Table 1). The results of QuantiFERON-TB Gold in-tube test (QFT; Cellestis Ltd, Carnegie, Australia) were positive in 10 persons (“QFT^+^ TB contacts”) and negative in 11 persons (“QFT^−^ TB contacts”, Table S2).

A group of healthy individuals included 15 participants with no records of *Mtb* exposure, negative results of QFT test and no clinical signs of TB (“*Mtb*-unexposed”; median age, 28 years; range, 19–71 years; 8 women; Table 1).

### Clinical and Radiographic Evaluations of TB Patients

Disease manifestations were evaluated and scored by independent clinicians and radiologists who were unaware of the results of immunological assays. Clinical disease severity was scored from 0 to 3 based on the extent of systemic intoxication symptoms (fatigue, sweating, fever, weight loss): 0– no symptoms; 1 - one to two symptoms without fever; 2 - several symptoms plus sub-febrile body temperature; 3 - several symptoms plus febrile temperature. Hematologic abnormalities were scored based on changes in erythrocyte sedimentation rate, leucocytosis, lymphopenia, left neutrophilic shift (0 - no abnormalities; 1 - one abnormality; 2 - two abnormalities; 3 - at least three severe abnormalities).

**Figure 1:**
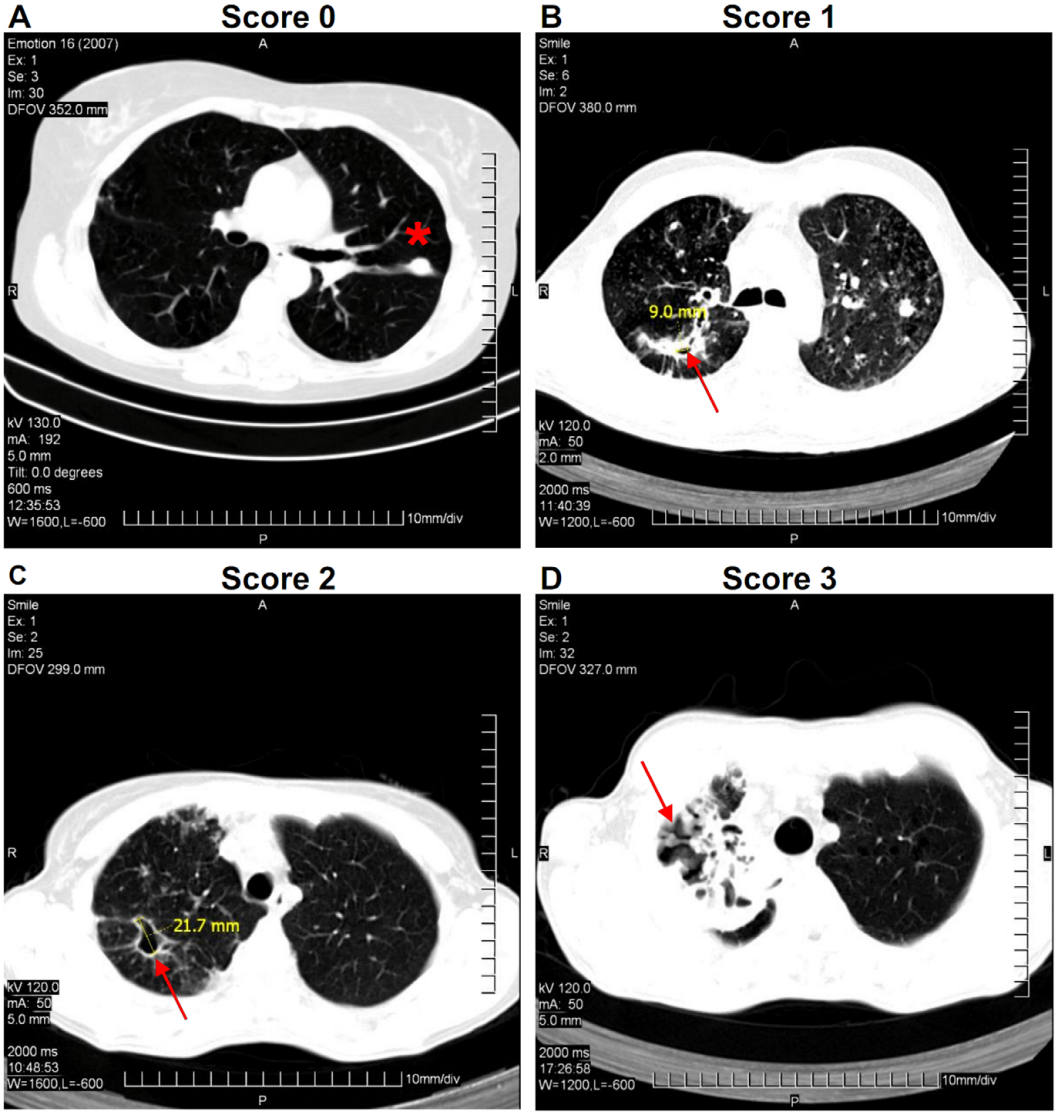
Evaluation of lung tissue destruction. Examples of X-ray computer tomograpy with different degrees of tissue destruction are shown. Lung destruction was evaluated based on the number and size of destructive (lucent) foci (see Methods for the details). A, score 0, no destruction; B, score 1, one small (<2 cm) destruction; C, score 2, one large (>2 cm) destruction; D, score 3, multiple lung destructions. Arrows, lung destructions. Asterisk, infiltrative focus without destruction.

TB extent, the form of lung TB pathology and the degree of lung tissue destruction were evaluated based on radiographic examinations. TB extent (the area affected by TB lesions) was scored as: 1 - one to three segments in different lobes; 2 - four or more segments in different lobes or one-two whole lobe(s); 3 - three lobes in different lungs; 4 - one whole lung or both lungs. The degree of lung tissue destruction was assessed based on the number and size of destructive (lucent) foci: 0 - no foci; 1 - one small (<2 cm diameter) focus; 2 - several small (<2 cm) foci or one large (≥2 cm) transparent focus; 3 - multiple foci of which at least one is large (≥2 cm, Fig. 1). The forms of lung TB pathology included: tuberculoma, TB infiltrate, cavitary TB, caseous pneumonia (Table 1, Table S1).

### CD27^low^ CD4 T Cell Analysis

**Figure 2:**
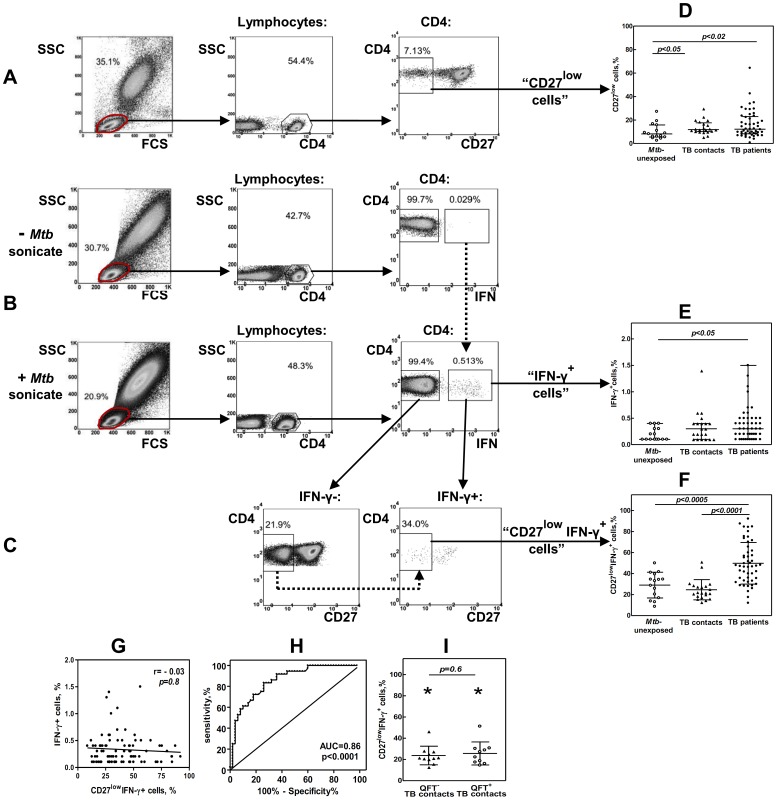
TB patients have increased percentages of CD27^low^IFN-γ^+^ CD4 T cells in their blood. A–C, Strategies for determining percentages of CD27^low^ (A), IFN-γ^+^ (B) and CD27^low^IFN-γ^+^ (C) CD4 T cells. A, CD27^low^ cells were gated within the total population of CD4^+^ T cells. B, To identify IFN-γ^+^ CD4 T cells, an aliquot of blood was stimulated with *Mtb* sonicate; another aliquote was left un-stimulated. During the analysis, the gates for IFN-γ^+^ cells in *Mtb*-stimulated samples were plotted based on *Mtb* un-stimulated samples (Fig. B, dotted line). To identify CD27^low^IFN-γ^+^ cells, the expression of CD27 was first analyzed in IFN-γ^−^ population. Because this population was always numerous, CD27^low^ and CD27^hi^ cells could be easily separated. The gates for CD27^low^ cells were then applied to IFN-γ^+^ population (C, dotted line). D–F, Percentages of CD27^low^ (D), IFN-γ^+^ (E), and CD27^low^IFN-γ^+^ (F) cells in TB patients (n = 50), TB contacts (n = 21) and *Mtb*-unexposed individuals (n = 15). G, Lack of correlation between the percentages of IFN-γ^+^ and CD27^low^IFN-γ^+^ cells in TB patients, TB contacts and *Mtb*-unexposed individuals (n = 86). H, ROC-curve of CD27^low^IFN- _γ_^+^ cell percentages for discriminating TB patients from healthy individuals (TB contacts and *Mtb*-unexposed). I, Percentages of CD27^low^IFN-γ^+^ cells in TB contacts with positive and negative results of QFT assay *p<0.0005 compared to TB patients.

Percentages of CD27^low^ lymphocytes were determined within: i) the total population of CD4 T cells (“CD27^low^” cells); (ii) *Mtb*-specific population of CD4 T cells (“CD27^low^IFN-γ^+^” cells). *Mtb*-specific lymphocytes were identified as CD4 T cells producing IFN-γ in response to stimulation with *Mtb*–specific antigens (“IFN-γ^+^” cells, Fig. 2A–C). For CD27^low^ cells, 200 ul of freshly isolated blood were stained with PerCP-Cy5.5 anti-CD4 and PE-anti-CD27 mAbs (BD Biosciences, San Jose, USA; e-bioscience, San Diego, USA; 10 min, room temperature). Red blood cells were lysed with BD FACS Lysing solution (BD Biosciences); the cells were washed and analyzed. Following analysis, the percentages of CD27^low^ cells within total population of CD4^+^ lymphocytes were determined (Fig. 2A). For CD27^low^IFN-γ^+^ cells, 0.5 ml of whole blood was diluted 1/1 in RPMI and cultured in the presence of *Mtb* sonicate (37°C, 5% CO_2_, 10 ug/ml protein of *Mtb* sonicate prepared as described earlier, [26]). The stimulation was performed within 5 hours of blood collection (preliminary analysis showed that this storage did not affect the results of the test). As a negative control, an aliquot of blood was always cultured without addition of *Mtb* sonicate. After 4 hours of culture, GolgiPlug (BD Biosciences) was added and cells were incubated for additional 14 hours. Cells were then stained with PerCP-Cy5.5-anti-CD4 and PE-anti-CD27 mAbs, treated with BD FACS Lysing solution and BD FACS Permeabilizing solution II, stained with APC-anti-IFN-γ mAbs (BD Biosciences), fixed and analyzed.

Following analysis, percentages of IFN-γ-producing cells were determined within the CD4^+^ population (Fig. 2B); percentages of CD27^low^ cells – within IFN-γ^+^ lymphocytes (Fig. 2C). The details of the gating strategy are presented in Fig. 2 (A–C. The validity of the gates for IFN-γ^+^ cells was confirmed by fluorescence minus one control (not shown). In *Mtb* sonicate un-stimulated samples, IFN-γ^+^ cells were largely absent, supporting that IFN-γ^+^ cells identified in stimulated samples represented *Mtb*-specific lymphocytes.

Absolute numbers of CD27^low^IFN-γ^+^ cells per one mL of blood were calculated as follows: ^N^CD27−IFN-γ+^ =  P^CD27− *^P^IFN-γ+ *^P^CD4+ *^N^lymph/(100)^3^, where ^P^CD27^−^ − percentages of CD27^low^ cells within IFN-γ^+^ cells; P_IFN-γ+_ − percentages of IFN-γ^+^ cells within CD4^+^ lymphocytes, P_CD4+._ − percentages of CD4^+^ cells within all lymphocytes, N_lymph_ – numbers of lymphocytes in 1 mL of blood (determined in hematology test on the day of analysis).

For lung and lymph node (LN) cell analysis, suspensions were prepared from samples of surgically resected lung tissue or lymph nodes. Cells were stimulated, stained and analyzed as described above. To compare pools of IFN-γ^+^ and CD27^low^IFN-γ^+^ cells in the lungs, lymph nodes and blood, absolute numbers of these lymphocytes per one million of acquired cells were calculated. All cells were analyzed using a BD Biosciences FACSCalibur with CellQuest (BD Biosciences) and FlowJo (Tree Star) softwares. Several reagents were kindly gifted to us by BD Biosciences.

### Statistical Analysis

In the text, if not indicated otherwise, medians, 25% and 75% percentiles are shown. Differences between unpaired and paired samples were analyzed using non-parametric Mann-Whitney and Wilcoxon tests. Sensitivity and specificity were determined by Receiver Operating Characteristic (ROC) curve analysis (GraphPad Software, Inc., San Diego, CA); cutoffs were selected based on the maximum sensitivity and specificity summary. Correlations were performed using Spearman analysis (GraphPad Software; program R, http://www.r-project.org). For multiple correlation analyses, p-value of 0.007 (7 variables) was considered significant to account for multiple testing. Best models were selected by multiple regression analysis using F-test for nested models and Akaike Information Criterion [27]–[29]. In bootstrapping analysis a pseudo dataset was created by re-sampling the data; 1000 simulations were performed and in each the best minimal model was selected using Akaike Information Criterion [28].

## Results

### TB Patients have Higher Percentages of *Mtb*-specific CD27^low^ CD4 T Cells in their Blood than *Mtb* Un-exposed Individuals and TB Contacts

Our previous studies in mice suggested that the degree of CD27^hi^→CD27^low^ differentiation may serve as an indicator of disease activity within *Mtb*-infected lungs. To test this hypothesis, in the present study we evaluated the degree of CD27^hi^→CD27^low^ differentiation of CD4 T cells in patients with pulmonary TB.

The differentiation status of CD4 T cells was evaluated by determining the percentages of CD27^low^ cells within the total population of CD4 T cells (“CD27^low^” cells) and within *Mtb*- specific subset of CD4 T cells (“CD27^low^IFN-γ^+^” cells). *Mtb-*specific cells were identified as CD4 T cells producing IFN-γ in response to stimulation with mycobacterial antigens (“IFN-γ^+^” cells).

In the first part of the study we compared the percentages of CD27^low^, IFN-γ^+^, and CD27^low^IFN-γ^+^ cells in the blood of TB patients (n = 50), TB contacts (n = 21) and *Mtb*- unexposed individuals (n = 15).

The percentages of CD27^low^ cells were higher in TB patients, relative to *Mtb*-unexposed individuals (12.2 [8.3–23.0] vs 8.2 [5.5–15.8], respectively; p = 0.03), but did not differ between TB patients and TB contacts (12.2 [8.3–23.0] vs 11.9 [10.1–17.6]; p>0.5; Fig. 2D). Similarly, the percentages of IFN-γ^+^ cells were higher in TB patients as compared to *Mtb*- unexposed individuals (0.3 [0.2–0.5] vs 0.1 [0.1–0.3]), but were similar in TB patients and TB contacts (0.3 [0.2–0.5] vs 0.3 [0.1–0.4]; p>0.5, Fig. 2E). The later finding is consistent with other studies, which revealed that IFN-γ assays were not useful for discriminating active TB disease and latent *Mtb* infection [11]. Thus, neither the expression of CD27 on bulk CD4 T lymphocytes, nor the frequency of *Mtb*-specific IFN-γ producing CD4 T cells, discriminated active TB from latent infection.

In contrast, the percentages of CD27^low^IFN-γ^+^ cells were significantly higher in TB patients (47.3 [33.2–63.6]) compared to either *Mtb*-unexposed participants (33.1 [16.8–39.5], p<0.0005) or TB contacts (21.8 [18.0–28.5], p<0.0001, Fig. 2F), which was in line with results published previously by Kern’s group [14]. The percentages of IFN-γ^+^ and CD27^low^IFN-γ^+^ cells were not statistically correlated (r = −0.03, p = 0.8, Fig. 2G). Thus, TB patients and TB contacts differed, not by the frequency of *Mtb*-specific CD4 T cells, but rather, by the degree of their differentiation.

ROC curve analysis confirmed that the percentages of CD27^low^IFN-γ^+^ cells (but not CD27^low^ or IFN-γ^+^ cells) provided a means to distinguish TB patients and TB contacts (82% sensitivity and 91% specificity, Table 2). Moreover, TB patients could be distinguished from all healthy participants (i.e., TB contacts and *Mtb*-unexposed individuals; 74% sensitivity and 83% specificity; Table 2, Fig. 2H). A cut off value for the percentages of CD27^low^IFN-γ^+^ cells that separated TB patients from all healthy participants with best specificity and sensitivity was 35.1%; this value was used in our further analyses as an upper limit of the norm.

**Table 2:** Discrimination of TB patients from different groups of healthy participants by the percentages of CD27^low^IFN-γ^+^ cells^1^.

Group of comparison	AUC	95% CI	p-value	Best cutoff, %	Sensitivity,%	Specificity,%	LR	OR
**All healthy participants (** ***Mtb*** **-unexposed and TB contacts)**	0.86	0.78–0.93	<0.0001	35.1	74	83	3.2	14.2
**All TB contacts**	0.89	0.81–0.97	<0.0001	31.2	82	91	5.0	41.5
**QFT^+^ TB contacts**	0.87	0.75–0.99	0.0003	31.2	82	90	5.0	41.5
**QFT^–^ TB contacts**	0.92	0.83–1.00	<0.0001	28.2	90	91	9.1	90
***Mtb*** **-unexposed**	0.80	0.69–0.92	0.0004	35.1	74	73	2.8	7.9

1 The percentages of CD27^low^IFN-γ^+^ cells were compared in TB patients and indicated groups of healthy participants. AUC, area under curve; CI, confidence interval; LR, likelihood ratio; OR, odds ratio.

Because some of TB contacts included in our study had positive results of QFT assay (QFT^+^, n = 10), while others were negative (QFT**^–^**, n = 11), we analyzed whether these two subgroups differed by the percentages of CD27^low^IFN-γ^+^ cells. The percentages were similar in both subgroups (p = 0.6); furthermore, the percentages did not differ from those observed in *Mtb*-unexposed individuals (p>0.2, Fig. 2I), but differed significantly from TB patients (p<0.0005). On ROC curve, similar cutoff values for the percentages of CD27^low^IFN-γ^+^ cells separated TB patients from QFT^+^ and QFT**^–^** contacts (Table 2). Overall, the percentages of CD27^low^IFN-γ^+^ cells were uniformly low in all healthy individuals and were significantly increased in patients with active TB. These results were in line with previous reports [14] and suggested that CD27^low^IFN-γ^+^ cells could serve as a measure of TB activity.

### In TB Patients, Accumulation of *Mtb*-specific CD27^low^ CD4 T Cells in Peripheral Blood is Associated with Lung Tissue Destruction

In comparison with *Mtb*-unexposed participants and TB contacts, TB patients had higher percentages of CD27^low^IFN-γ^+^ cells. Yet, these percentages varied substantially among individual patients (i.e., from 12 to 92%, Fig. 2F). Previously, several authors had also observed variability among TB patients in the percentages of CD27^low^ *Mtb*-specific CD4 T cells [14], [30]. However, the mechanisms underlying this variability and its relevance to TB monitoring remained unaddressed.

To start addressing these mechanisms, we analyzed whether the percentages of CD27^low^IFN-γ^+^ cells were associated with different TB manifestations. The following characteristics of TB disease were taken into account: duration of TB disease, the presence of *Mtb* in sputum smear/culture, *Mtb* drug resistance, the form of lung pathology, the extent of pulmonary TB, the degree of lung tissue destruction, disease severity (assessed separately based on clinical symptoms and hematologic abnormalities). All characteristics were evaluated blindly by physicians, radiologists and microbiologists who were unaware of the results of immunological assays.

**Figure 3:**
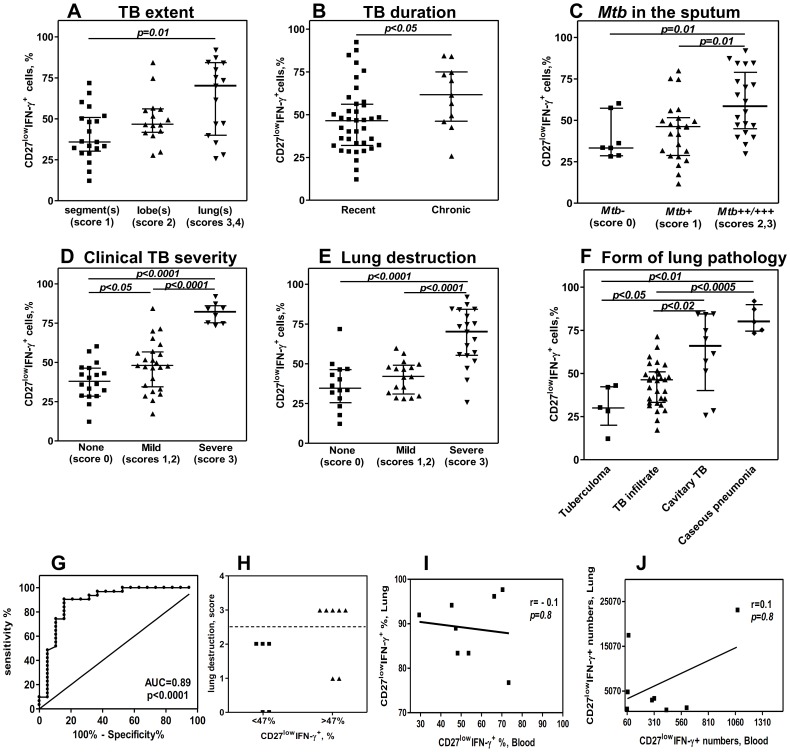
Association between blood CD27^low^IFN-γ^+^ cells and different manifestations of TB disease. A–F, Percentages of CD27^low^IFN-γ^+^ cells in TB patients (n = 50) grouped based on different characteristics of TB disease. For multiple (seven) parameter testing, p- value <0.007 was considered significant. G, ROC curve for discriminating TB patients with high (score 3) and low (scores 0–2) degrees of lung tissue destruction (n = 50). H, The degree of lung tissue destruction in patients with “low” (<47%) and high (>47%) percentages of CD27^low^IFN-γ^+^ CD4 T cells (n = 12, validation analysis). I, J, Lack of correlation between the percentages (I) and the numbers (J) of CD27^low^IFN-γ^+^ cells in the lungs and in the blood of TB patients (n = 8). Indicated are numbers of CD27^low^IFN-γ^+^ cells per 1 million of acquired cells.

As a first step, we divided TB patients into groups with different levels of a given factor and compared the percentages of CD27^low^IFN-γ^+^ cells in these groups. We found that the percentages of CD27^low^IFN-γ^+^ cells did not differ between groups of patients with different TB extent, with different disease durations, and with high and low content of *Mtb* in the sputum (p = 0.01, p = 0.04, and p = 0.01, respectively, insignificant for multiple parameter testing; Fig. 3A–C). In contrast, the percentages of CD27^low^IFN-γ^+^ cells differed significantly between groups of patients having different degrees of lung destruction, clinical disease severity, and different forms of lung TB pathology (p<0.007, Fig. 3D–F).

Next, we looked at correlations between the percentages of CD27^low^IFN-γ^+^ cells and magnitude of a given factor. In this analysis, factors that correlated most significantly with the percentages of CD27^low^IFN-γ^+^ cells were: lung tissue destruction (r = 0.65, p = 2.7×10^−7^), clinical disease severity (r = 0.63, p = 7.7×10^−7^), hematologic abnormalities (r = 0.49, p = 3×10^−4^). The presence of *Mtb* in the sputum and TB extent correlated less strongly (r = 0.41, p = 0.003. and r = 0.42, p = 0.003, respectively; Table 3). The correlations between the percentages of CD27^low^IFN-γ^+^ cells and *Mtb* drug resistance or TB duration were insignificant (p = 0.01 and p = 0.04, respectively, insignificant when adjusted for multiple comparisons). The correlation between the percentages of CD27^low^IFN-γ^+^ cells and the form of lung pathology was highly significant (r = 0.63, p = 8.7×10^−7^), but it was likely secondary to lung destruction: it appeared only when higher scores were assigned to more destructive TB forms (i.e., tuberculoma – score 1; TB infiltrate – score 2; cavitary TB – score 3; caseous pneumonia – score 4); within a group of patients with the same form of lung TB pathology (e.g., TB infiltrate) the percentages of CD27^low^IFN-γ^+^ cells correlated with lung destruction (r = 0.45, p<0.01).

**Table 3:** Correlation between TB manifestations and percentages of CD27^low^IFN-γ^+^ CD4 T cells in the blood of TB patients.

Factors		Simple correlation (Spearman)^1,2^		Multiple linear regression^1,3^			
	n	rho	p-value	Estimate	Std. error	t-value	p-value
**Lung tissue destruction**	50	**0.65**	**2.7×10** ^−**7**^	**4.439**	**2.127**	**2.087**	**0.043**
	62	**0.66**	**5×10** ^−**9**^	**5.8296**	**2.0309**	**2.870**	**0.00578**
**Clinical TB severity**	50	**0.63**	**7.7×10^−7^**	**8.579**	**2.458**	**3.490**	**0.001**
	62	**0.55**	**3.2×10^−6^**	**6.0474**	**2.5473**	**2.374**	**0.02105**
**Hematologic abnormalities**	50	**0.49**	**3.0×10^−4^**	1.258	2.200	0.572	0.570
	62	**0.5**	**3.4×10^−5^**	1.7958	2.2448	0.800	0.4270
**Sputum ** ***Mtb*** ** positivity**	50	**0.41**	**0.003**	1.358	2.505	0.542	0.590
	62	**0.36**	**0.004**	−0.2399	2.5495	−0.094	0.92538
**TB extent**	50	**0.42**	**0.003**	0.447	2.188	0.204	0.839
	62	**0.38**	**0.002**	1.1142	2.0340	0.548	0.5860
***Mtb*** ** multi-drug resistance**	50	0.37	0.01	NA^4^	NA	NA	NA
	62	0.28	0.04	NA	NA	NA	NA
**TB duration**	50	0.29	0.04	NA	NA	NA	NA
	62	0.28	0.02	NA	NA	NA	NA

1 Analysis was initially performed in 50 patients. Subsequently, 12 patients from validation cohort were added (n = 62), mainly to check the consistency of the results. The results obtained in both cohorts are shown.

2 Simple correlation analysis selects five major predictors for the accumulation of IFN-γ^+^CD27^low^ cells in the blood of TB patients (highlighted in bold). For TB duration and *Mtb* multi-drug resistance p-values were >0.007 (insignificant for multiple (seven) parameter testing); these factors were not included in multiple linear regression analysis. rho, Spearman coefficient, p, significance value of the test.

3 Multiple linear regression identified lung tissue destruction and clinical TB severity as the main correlates for the accumulation of CD27^low^IFN-γ^+^ cells in the blood of TB patients (highlighted in bold).

4 NA, not included in multiple linear regression analysis.

While the percentages of CD27^low^IFN-γ^+^ cells correlated strongly with several TB characteristics (e.g., TB severity, lung destruction), the numbers of CD27^low^IFN-γ^+^ cells (per ml of blood) did not correlate significantly with either of the factors analyzed (Table S3). This result can be explained by the fact that percentages of CD27^low^IFN-γ^+^ cells represent a simple parameter that mirrors only the degree of CD27^+^→CD27^−^ CD4 T cell differentiation. In contrast, the number of CD27^low^IFN-γ^+^ cells represents a complex parameter that, besides the percentages of CD27^low^IFN-γ^+^ cells, depends on the total number of lymphocytes, CD4 T cells, and IFN-γ producing cells. The course of TB disease may affect these parameters in different ways (e.g., stimulate CD27^+^→CD27^−^ differentiation and increase CD27^low^IFN-γ^+^ cell frequency, but induce lymphopnenia and decrease total numbers of lymphocytes and their subsets).

Because the percentages of CD27^low^IFN-γ^+^ cells correlated significantly with several different manifestations of TB disease, we next addressed, which of the identified correlates were most important for determining the percentages of CD27^low^IFN-γ^+^ cells in the blood of TB patients. In multiple regression analysis, high percentages of CD27^low^IFN-γ^+^ cells correlated significantly with lung destruction and clinical TB severity (Table 3). In several additional analyses (F-test for nested models and model selection using Akaike Information Criterion), lung destruction, clinical disease severity and hematologic abnormalities, or lung destruction and clinical disease severity alone predicted the percentages of CD27^low^IFN-γ^+^ cells (Table 4). Any of these factors taken separately did not predict the percentages of CD27^low^IFN-γ^+^ cells well, suggesting that lung tissue destruction and TB severity were important and both were needed to explain increased percentages of CD27^low^IFN-γ^+^ cells in TB patients. When a method for random data resampling was used [28], lung destruction and clinical TB severity also appeared most often as factors determining high percentages of CD27^low^IFN-γ^+^ cells in TB patients (13% and 15% of cases, respectively).

**Table 4:** Selection of minimal model to explain variability in the percentages of CD27^low^IFN-γ^+^ cells between TB patients.

Factor(s)	F test (p), n = 50^2^	F test (p), n = 62^2^
Minimal model with single factor^1^		
**Lung tissue destruction**	0.01618	0.02315
**Clinical TB severity**	**0.1017**	0.01493
**Hematologic abnormalities**	4.6×10^−5^	2.8×10^−4^
**Sputum ** ***Mtb*** ** positivity**	2.7×10^−6^	2.0×10^−6^
**TB extent**	1.7×10^−5^	1.6×10^−5^
**Minimal models with several factors** 1		
**Lung destruction + clinical TB severity + hematologic abnormalities**	**0.7972**	**0.8571**
**Lung destruction + clinical TB severity** 3	**0.7701**	**0.7804**
**Lung destruction + hematologic abnormalities**	0.0062	**0.0890**
**TB severity + hematologic abnormalities**	**0.1178**	0.0248

1 Best minimal models are those that differ insignificantly from the full model (highlighted in bold).

2 Analysis was initially performed in 50 patients. Subsequently, 12 patients from validation cohort were added (n = 62), mainly to check the consistency of the results. In both cohorts, lung destruction and clinical disease severity predicted best the accumulation of CD27^low^IFN-γ^+^ cells.

3 In Akaike Information Criterion, this combination was the best minimal model to predict the accumulation of CD27^low^IFN-γ^+^ cells in the blood ( Δ_n = 50_ = 4.7; Δ_n = 62_ = 4.8).

Because the degree of lung tissue destruction appeared as a good correlate of the percentages of CD27^low^IFN-γ^+^ cells and because destruction of pulmonary matrix is an important pathogenic factor that causes TB progression, morbidity, and bacillary spread, we next asked whether evaluation of CD27^low^IFN-γ^+^ cells could be used as a means to estimate pulmonary destruction.

On the Receiver Operating Characteristic (ROC) curve, a cutoff of 47% of CD27^low^IFN-γ^+^ cells appeared as a good separator of TB patients with severe lung destruction (score 3) and other patients (scores 0–2) (AUC = 0.89, p<0.0001, odds ratio = 20.7, likelihood ratio = 7.1, sensitivity, 89%, specificity, 74%; Fig. 3G). Thus, in our study a cutoff of 35.1% of CD27^low^IFN-γ^+^ cells discriminated TB patients and healthy participants; a cutoff of 47% predicted the degree of lung destruction in TB patients.

To validate these results, a group of additional 12 patients was enrolled. In these patients, we determined the percentages of CD27^low^IFN-γ^+^ cells and compared them to 35.1 and 47% thresholds. Disease threshold (35.1%) was exceeded in 9 of 12 patients (data not shown); therefore, the test identified TB patients with 75% sensitivity, which is close to the results obtained in the main group (74%). Severe lung destruction threshold (47%) was exceeded in 7 patients. As judged by the lung radiograms, 5 of these 7 patients had severe destructive lesions. In 5 of 12 patients, the percentages of CD27^low^IFN-γ^+^ cells were below the 47% threshold. As judged by the lung radiograms, none of these 5 patients had severe destructive lesions (Fig. 3H), which is again consistent with our findings in the main group. Overall, these results supported our hypothesis that analysis of CD27^low^IFN-γ^+^cells circulating in peripheral blood can be used to evaluate severity of destructive processes ongoing in the lungs during TB.

### At the Site of Lung *Mtb* Infection, the Majority of *Mtb*-specific CD4 T Lymphocytes are CD27^low^

Our previous studies in mice demonstrated that during *Mtb* infection, CD27^low^ CD4 T cells accumulate preferentially in the lungs and can be generated from CD27^hi^ precursors in the lungs [25]. We proposed, therefore, that variability in the percentages of CD27^low^IFN-γ^+^ cells in blood of TB patients was due to differences in the generation of CD27^low^ CD4 T cells in the lungs. To address this question, we analyzed the frequencies and numbers of CD27^low^IFN-γ^+^ and IFN-γ^+^ cells in the lungs and blood of eight patients who had undergone lung surgery.

In all patients, the percentages and numbers of IFN-γ^+^ and CD27^low^IFN-γ^+^ cells in the lungs were significantly higher than in the blood (p<0.002; Table S4), supporting that *Mtb*-specific (IFN-γ^+^) and highly differentiated *Mtb*-specific (CD27^low^IFN-γ^+^) CD4 T cells accumulated preferentially in the lungs.

Among the patients, the percentages and numbers of IFN-γ^+^ cells, and the numbers of CD27^low^IFN-γ^+^ cells in the lungs varied (5–31-fold). In contrast, the percentages of CD27^low^IFN-γ^+^ cells in the lungs were uniformly high (>76% of all IFN-γ^+^ cells), indicating that the majority of *Mtb*-specific lung CD4 T cells were highly differentiated (Table S4). The percentages of blood CD27^low^IFN-γ^+^ cells differed among the patients (30–74%). No significant correlation was observed between the percentages of CD27^low^IFN-γ^+^ cells residing in the lungs and circulating in the blood (Table S4, Fig. 3, I, J), indicating that variability in the percentages of CD27^low^IFN-γ^+^ cells in blood of TB patients was not due to differences in the generation (accumulation) of these cells in the lungs. A similar lack of correlation was found for IFN-γ^+^ cells (Table S4). Furthermore, the percentages of CD27^low^IFN-γ^+^ or IFN-γ^+^ cells in the lungs did not correlate significantly with any of the criteria used to categorize TB disease (i.e., disease severity, lung destruction et al., Table S4). In contrast, the percentages of CD27^low^IFN- γ^+^ cells in the blood were well correlated with lung destruction (r = 0.88, p = 0.01, Table S4), which was consistent with our results obtained in the main group.

In two patients, regional lymph nodes were resected during the surgery. The percentages and numbers of CD27^low^IFN-γ^+^ cells were lower in the LNs as compared to the lungs (per 1 million of acquired cells, Table S4). This is in line with our previous findings in mice where we found low percentages of CD27^low^ and IFN-γ^+^ CD4 T cells in the lymph nodes and their preferential accumulation in the lung tissue [24], [25].

Thus, we observed (i) a preferential accumulation of CD27^low^ *Mtb*-specific CD4 T cells in the lungs; (ii) a poor correlation between the magnitude of CD27^low^ *Mtb*-specific CD4 T cell response in the lungs and in the blood; (iii) that lung tissue destruction was correlated with the percentages of CD27^low^IFN-γ^+^ cells present in the blood, but not in the lungs. These observations suggested that evaluation of CD27^low^ *Mtb*-specific CD4 T cells circulating in the blood may provide a means to assess pulmonary destruction during TB.

### Following TB Treatment, the Percentages of *Mtb*-specific CD27^low^ CD4 T Cells Decline Parallel to Reduction/cessation of Lung Tissue Destruction

**Figure 4:**
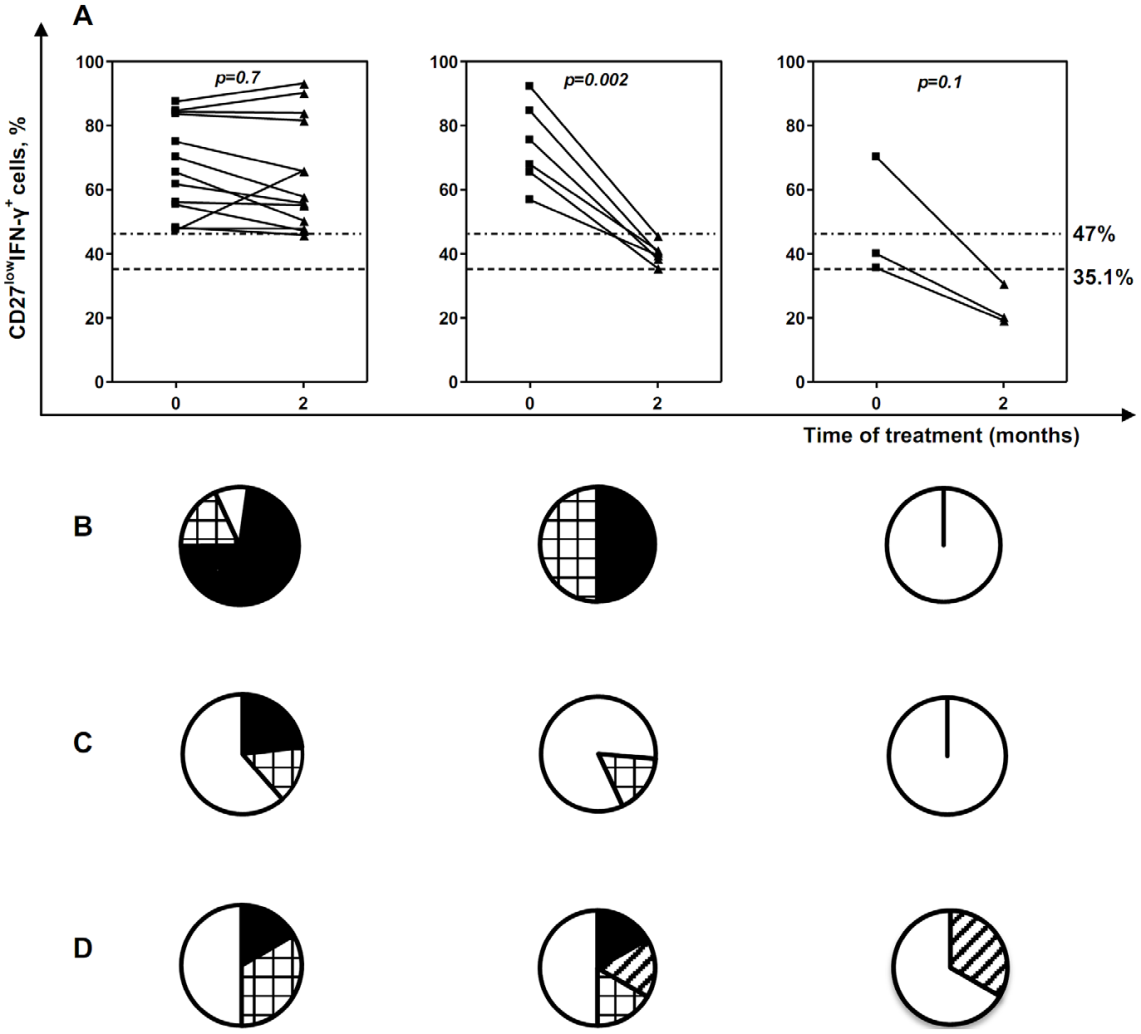
Following anti-TB therapy, CD27^low^IFN-γ^+^ cells decline parallel to reduction/repair of lung destruction. A, Percentages of CD27^low^IFN-γ^+^ cells were determined at the start of treatment and two months later. Depending on the results of “IFN-γ/CD27” assay, patients were divided into three groups: in “CD27^low^- high” group, initially elevated percentages of CD27^low^IFN-γ^+^ cells did not decline following 2-mo therapy; in “CD27^low^ – reduced” group, percentages of CD27^low^IFN-γ^+^ cells declined to become below the 47% threshold, but remained above 35.1% threshold (upper limit of norm); in “CD27^low^ - normalized” group, percentages of CD27^low^IFN-γ^+^ cells declined to become below the 35.1% threshold. Dotted lines show 35.1 and 47% thresholds. B–D, diagrams showing changes in lung destruction (B), sputum *Mtb*-positivity (C) and clinical TB severity (D) for each group of patients following 2-mo therapy. Closed segments, no improvement; squared segments, reduction of lung destruction, clinical symptoms or numbers of *Mtb* in the sputum; open segments, normalization (repair of lung destruction, conversion of sputum assay, disappearance of intoxication symptoms), striped segments – no abnormalities at the start of the treatment.

Next we investigated whether and how the percentages of circulating CD27^low^IFN-γ^+^ CD4 T cells change following TB treatment. Analyses were performed at the start of treatment and 2 months later. Totally, 22 patients with initially elevated percentages of CD27^low^IFN-γ^+^ cells (above 35.1%) were included in the analysis. Based on changes in the percentages of CD27^low^IFN-γ^+^ cells, the patients were divided into three groups (Fig. 4A). For each patient, responsiveness to treatment in terms of repair/reduction of pulmonary destruction, reduction of disease severity and conversion of sputum assay were evaluated blindly and compared to the results of “IFN-γ/CD27” assay.

In 13 of 22 patients, the percentages of CD27^low^IFN-γ^+^ cells did not decline by the end of 2- mo therapy or declined only slightly to remain above 47% threshold (threshold that in our study discriminated TB patients with severe and mild lung tissue destruction; “CD27-high” group). Radiologically, the majority of patients (10 patients, 77%) retained severe lung destruction (score 3) till the end of 2-mo therapy (Fig. 4B). In four of these patients foci of severe destruction persisted during at least 4 months of treatment (follow-up period).

In 6 patients, CD27^low^IFN-γ^+^ cell percentages reduced significantly (by >30% of the initial value) and reached less than 47% threshold level, although they did not normalize completely (i.e., remained >35.1% threshold, “CD27-reduced” group). Radiologically, reduction of lung tissue destructions by the end of 2-mo therapy was registered in 3 patients (50%), and by the end of 4-mo therapy - in all followed-up patients (n = 6).

In 3 patients, the percentages of CD27^low^IFN-γ^+^ cells decreased to achieve normal values by the end of 2-mo therapy (<35.1%, “CD27-normalized” group). Radiologically, in all three patients lung destructive foci repaired by the end of 2-mo therapy.

Thus, high percentages of CD27^low^IFN-γ^+^ cells during therapy were associated with persistence of tissue destruction, while reduction or normalization of CD27^low^IFN-γ^+^ cell percentages was associated with the ongoing or completed repair of the damaged tissue. Overall, there was a significant correlation between a decline in the percentages of blood CD27^low^IFN-γ^+^ cells and repair of lung destruction (r = 0.54, p<0.01).

In contrast to lung destruction, no significant correlation was revealed between changes in the percentages of CD27^low^IFN-γ^+^ cells and conversion of sputum assay or clinical TB improvement (r = 0.23, p = 0.31 and r = 0.22, p = 0.31, respectively). These factors improved prior to lung tissue repair; their improvement was observed even in patients with persistently high percentages of CD27^low^IFN-γ^+^ cells (Fig. 4C–D).

Overall, in our study, blood CD27^low^IFN-γ^+^ cells appeared as a measure of *Mtb* infection activity in the lungs: increased percentages of these cells distinguished TB patients from latently infected individuals; in TB patients, extremely high percentages of CD27^low^IFN-γ^+^ cells were indicative of severe lung tissue destruction; following TB treatment, a decrease in the percentages of CD27^low^IFN-γ^+^ cells was predictive of lung tissue repair.

## Discussion

In this study we have demonstrated that: (i) active TB induces a large increase in the highly differentiated CD27^low^ subset of *Mtb*-specific CD4 T cells in the lungs and in peripheral blood; (ii) an increase in the percentages of these cells in the blood during TB is associated with lung tissue destruction and disease severity; (iii) evaluation CD27^low^ *Mtb*-specific CD4 T cells provides a valuable means to assess TB activity, the degree of *Mtb*-induced lung destruction and lung tissue repair following TB treatment.

CD27 is a member of the TNF-receptor superfamily. Lack of CD27 expression on T lymphocytes marks functionally mature highly differentiated effector T cells [20], [21], [23], [24]. Our data on high percentages of CD27^low^ cells within a population of *Mtb*-specific CD4 T lymphocytes in TB patients, indicate that that there is no deficiency in the generation of CD4 effectors during severe TB. Instead, active TB is accompanied by a high degree of CD4 T cell differentiation, which is in line with our previous data in mice showing that *Mtb* infection drives CD27^hi^→CD27^low^ differentiation of effector CD4 T cells [25]. Our results correspond well to the results by Kern’s group [14] who first described an increase in the frequency of CD27^low^ *Mtb*-specific CD4 T cells in TB patients and suggested that evaluation of these cells can be used to discriminate TB patients from latently infected individuals. It should be noted that a cutoff discriminating TB patients and healthy individuals in our study (35.1%) was lower than that reported by Kern’s group (49%). The most likely explanation relies on methodological differences (stimulation of whole blood in our study *versus* stimulation of PBMC in the study by Kern’s group) and/or the differences in patients’ selection. Indeed, in the study by Kern’s group, patients with several lesions and at least one lesion larger than 3 cm diameter were enrolled; in our study, patients with single lung lesion and no signs of tissue destruction (i.e. patients in whom TB diagnostics is most problematic) were also included. As shown in our study, the latter patients have relatively low percentages of CD27^low^IFN-γ^+^ cells, and their inclusion in the analysis could lower the threshold discriminating TB patients from healthy participants. The major focus of the current study is the analysis of CD27^low^ response in TB patients. We found that at the site of *Mtb* infection in the lung, most *Mtb*-specific CD4 T lymphocytes (>76%) were CD27^low^. These results were obtained in all analyzed patients and supported a propensity of CD27^low^ cells for lung tissue location documented previously in different pathological conditions in mice and humans [25], [31]–[34].

In contrast to the lungs, the percentages of CD27^low^IFN-γ^+^ cells in the blood varied among the patients. There was no significant correlation between the percentages of these cells in the blood and in the lungs, but a significant correlation was found between the percentages of CD27^low^IFN-γ^+^ cells in the blood and lung tissue destruction and TB severity. The percentages of CD27^low^IFN-γ^+^ cells in the lung did not correlate with lung destruction or TB severity. The reasons for discordance between lung and blood CD27^low^IFN-γ^+^ cells and mechanisms associating blood CD27^low^IFN-γ^+^ cells, lung destruction and TB severity are not clear.

Effector T cells are thought to be generated in the LNs and migrate to peripheral sites via the circulation. We have shown previously that CD27^low^ CD4 effectors can be generated directly in the lungs from their CD27^hi^ effector precursors [25]. Therefore, high percentages of CD27^low^IFN-γ^+^ cells in the blood of patients with severe TB can result from their highly efficient generation in the LNs (e.g., due to extensive antigenic/inflammatory stimulation) and/or preferential retention in the circulation (e.g., due to highly inflammatory milieu). Uniformly high percentages of CD27^low^IFN-γ^+^ cells at the site of lung *Mtb* infection may be due to their efficient local formation. These scenarios explain an association between CD27^low^IFN-γ^+^ cells and TB severity, but do not explain why the percentages of blood CD27^low^IFN-γ^+^ cells did not depend on TB extent, or why they were tightly associated with lung destruction. There are several possible explanations for the later association.

First, lung destruction and accumulation of CD27^low^IFN-γ^+^ cells in the circulation may represent independent manifestations of severe TB. Second, CD27^low^IFN-γ^+^ cells generated in high quantities during severe TB may directly contribute to tissue destruction, e.g., by producing proinflammatory factors or stimulating inflammatory reactions in phagocytic cells. Indeed, in mice CD27^low^ CD4 T cells mediate these functions more efficiently than CD27^hi^ effectors (our unpublished observations); a role for metalloproteinases and proinflammatory cytokines in lung matrix destruction has been directly demonstrated [35], [36]; in other pathological conditions, CD27^low^ T cells have been associated with poor disease outcome [34]. However, these scenarios do not answer a question why blood but not lung CD27^low^IFN-γ^+^ cells were associated with lung tissue damage. They also do not explain why the percentages, but not the numbers of cells were associated with TB severity and lung destruction.

In a third hypothesis, accumulation of CD27^low^IFN-γ^+^ cells in blood is a result of lung destruction. In this hypothesis, CD27^low^ CD4 T cells represent a lung tissue resident population and normally have low capacity for tissue exit resembling recently described lung-retentive memory cells [37]. Once the architecture of the lung tissue and/or lung vessels is disrupted, CD27^low^ lymphocytes acquire the capacity to enter the circulation. This may become possible due to mechanical reasons or to a high local production of factors (e.g., proteases) altering cell adhesion to lung parenchyma. Undoubtedly, all described mechanisms may cooperate to promote the accumulation of CD27^low^IFN-γ^+^ cells in the circulation. Exact mechanisms associating high degree of blood CD4 T cell differentiation and lung tissue destruction are yet to be determined.

In conclusion, this study documents that there is no deficiency in the differentiation of effector CD4 T cells during severe pulmonary TB. The study for the first time reveals an association between CD27^low^ *Mtb*-specific CD4 T cells and lung tissue destruction and suggests an immunological assay to evaluate lung destruction and its repair following TB therapy (processes that until now could have been monitored only by X-ray examination). Finally, the study raises fundamental questions on mechanisms associating peripheral tissue destruction and differentiation status of circulating effector lymphocytes.

## Supporting Information

Table S1 **Baseline characteristics of patients included in this study.** ^1^In all patients, immunological analysis of blood cells was performed at the beginning of treatment. Initial analysis was performed in 50 patients (“main” group). The results were validated in 12 patients (“validation” group). In some patients, additional analysis was performed 2 months following the treatment (“dynamic” group). In patients undergoing lung surgery, blood and lung cells were analyzed on the day of surgery (“Surgery” group). ^2^+++, ≥10 AFB per view field; ++, 1–10 AFB per view field; +, 10−99 AFB per 100 view fields or positive result of sputum culture/BACTEC; -, no *Mtb* identified in sputum smear or culture. ^3^S, drug sensitive; MDR, multidrug-resistant; XDR, extensively drug-resistant; NA, not applicable (no *Mtb* identified in the culture). ^4^Indicated are areas (lobes, segments) of lungs affected by TB infection. Lobes: UR, upper right; MR, middle right; LR, low right; UL, upper left; LL, low left; S1, S2, etc., segments of lobes. ^5^Indicated are numbers of destructive foci. Small, the size (diameter) of the focus is <2 cm. For multiple foci the size of the largest is shown in parentheses (if ≥2 cm); system, system of communicating destructive foci. ^6^Responsiveness to TB treatment was assessed 2 months following the therapy based on the results of X-ray examination (reduction of lung tissue infiltration; reduction/repair of lung destruction), hematology test and clinical follow-up. 0, no positive dynamics; +, reduction of lung tissue infiltration/destruction, hematologic abnormalities and clinical TB severity; ++, consolidation of pulmonary infiltration, repair of lung destruction, normalization of hematologic abnormalities and clinical severity; N, parameters were initially normal. + (surgery), positive dynamics was observed in response to lung surgery.
(PDF)

Table S2 **Characterization of TB contacts and** ***Mtb*** **-unexposed participants.** ^1^Time of work in TB hospital (years). ^2^Not applicable.
(PDF)

Table S3 **Lack of correlation between TB manifestations and numbers of CD27^low^** **IFN-γ^+^ cells in the blood of TB patients.** None of the analyzed factors correlated significantly with the numbers of CD27^low^IFN-γ^+^ cells in the blood of TB patients. Initial analysis was performed in 50 patients. Subsequently, 12 patients from validation cohort were added (n = 62). In both cohorts, similar results were obtained (shown are results obtained in 50 patients). rho, Spearman coefficient, p, significance value of the test.
(PDF)

Table S4 **Percentages and numbers of IFN-γ^+^ and CD27^low^ IFN-γ^+^ cells in the lungs and in blood of surgery operated patients.** Blood and lung cells were analyzed on the day of lung surgery. Indicated are percentages and numbers (per 1 million of acquired cells) of IFN-γ^+^ and CD27^low^IFN-γ^+^ cells. ^1^Fold differences between maximal and minimal values in the group. ^2^Correlations were analyzed between: the indicated cells in the lungs and blood; lung destruction and indicated cells; TB severity and indicated cells.
(PDF)
