# Supplementary material for: Mtb-Specific CD27low CD4 T Cells as Markers of Lung Tissue Destruction during Pulmonary Tuberculosis in Humans
Source: PLoS One. 2012 Aug 24;7(8):e43733. doi: 10.1371/journal.pone.0043733 (PMC3427145; doi:10.1371/journal.pone.0043733)
Supplement: Table S1 — Baseline characteristics of patients included in this study. 1In all patients, immunological analysis of blood cells was performed at the beginning of treatment. Initial analysis was performed in 50 patients (“main” group). The results were validated in 12 patients (“validation” group). In some patients, additional analysis was performed 2 months following the treatment (“dynamic” group). In patients undergoing lung surgery, blood and lung cells were analyzed on the day of surgery (“Surgery” group). 2+++, ≥10 AFB per view field; ++, 1–10 AFB per view field; +, 10−99 AFB per 100 view fields or positive result of sputum culture/BACTEC; -, no Mtb identified in sputum smear or culture. 3S, drug sensitive; MDR, multidrug-resistant; XDR, extensively drug-resistant; NA, not applicable (no Mtb identified in the culture). 4Indicated are areas (lobes, segments) of lungs affected by TB infection. Lobes: UR, upper right; MR, middle right; LR, low right; UL, upper left; LL, low left; S1, S2, etc., segments of lobes. 5Indicated are numbers of destructive foci. Small, the size (diameter) of the focus is <2 cm. For multiple foci the size of the largest is shown in parentheses (if ≥2 cm); system, system of communicating destructive foci. 6Responsiveness to TB treatment was assessed 2 months following the therapy based on the results of X-ray examination (reduction of lung tissue infiltration; reduction/repair of lung destruction), hematology test and clinical follow-up. 0, no positive dynamics; +, reduction of lung tissue infiltration/destruction, hematologic abnormalities and clinical TB severity; ++, consolidation of pulmonary infiltration, repair of lung destruction, normalization of hematologic abnormalities and clinical severity; N, parameters were initially normal. + (surgery), positive dynamics was observed in response to lung surgery. (PDF) [file pone.0043733.s001.pdf]

Table S1. Baseline characteristics of patients included in the study.

#	Sex	Age	Group of patients[1]				TB duration	Forms of lung TB pathology	*Mtb* in the sputum[2]	*Mtb* drug resistance[3]	TB extent[4]		Lung destruction[5]		Hematology test scores	Clinical TB severity scores	Responsiveness to TB treatment[6]			
			Main	Validation	Dynamic studies	Surgery					Affected areas of the lung	Scores	Numbers of destructive foci (size, cm)	Scores			Infiltration (X-ray)	Destruction (X-ray)	Hematology test	Clinical TB severity
1	f	20	+				Recent	TB infiltrate	-	ND	UR: S_1,S_2	1	multiple, small	2	0	0	+	N	N	N
2	m	27	+				Recent	TB infiltrate	+	MDR	UL: S_{1+2}	1	one small	1	1	1	++	++	++	++
3	m	25	+				Recent	Cavitary TB	-	ND	UR: S_3	1	one small	1	0	0	++	++	N	N
4	f	41	+				Recent	TB infiltrate	++	DR	UR: S_3; LR: S_6	1	none	0	2	2	+	N	++	+
5	f	25	+				Recent	Cavitary TB	-	ND	LR: S_6	1	multiple (2.0 x 4.0)	3	1	2	+	+	++	++
6	f	30	+				Recent	TB infiltrate	-	ND	UR: S_1,S_2	1	none	0	1	0	++	N	++	N
7	f	37	+				Recent	TB infiltrate	+	s	UR, LR, LL	3	one (2.2 x 1.4)	2	2	0	+	+	+	N
8	f	28	+				Recent	TB infiltrate	-	s*	LR: S_{10}; MR: S_5	1	none	0	1	1	+	N	++	++
9	m	20	+				Recent	TB infiltrate	+	s	LR: S_7, S_8, S_9; LL: S_6	2	two small	2	2	2	++	+	++	++
10	m	40	+				Recent	TB infiltrate	+++	MDR	UR	2	none	0	3	1	+	N	0	++
11	f	23	+				Recent	Tuberculoma	++	s	UR: S_1,S_2; LR: S_6; LL: S_6	2	two small	2	0	2	+	+	N	++
12	f	25	+				Recent	TB infiltrate	+	MDR	LR: S_6	1	none	0	0	1	+	N	N	++
13	m	40	+				Chronic	Tuberculoma	+++	MDR	UL; UR	2	none	0	0	0	+	N	N	N
14	f	40	+				Recent	Cavitary TB	+	MDR	UL: S_{1+2},S_3	1	two small	2	2	1	+	+	0	0
15	f	31	+				Chronic	TB infiltrate	+	MDR	LL	2	none	0	1	0	+	N	++	N
16	f	32	+				Recent	TB infiltrate	+	s	UL: S_{1+2}	1	one small	1	0	0	+	++	N	N
17	m	27	+				Recent	Tuberculoma	+	s	UR: S_1,S_2; MR; LL	2	multiple, small	2	1	0	+	0	++	N
18	f	27	+				Chronic	Caseous pn.	+++	XDR	both lungs	4	multiple, system (9.0 x 7.0)	3	3	3	0	0	0	0
19	f	22	+				Recent	TB infiltrate	+	s	UL: S_{1+2}; LL: S_6	1	multiple small	2	0	0	+	+	N	N
20	f	57	+				Recent	TB infiltrate	+	s	UR: S_1,S_2; UL: S_{1+2}; LL: S_6	2	none	0	1	0	+	N	0	N
21	f	39	+				Recent	TB infiltrate	+	s	UR: S_1,S_2; LR: S_6	1	one small	1	3	1	+	N	++	++
22	m	27	+				Chronic	Cavitary TB	+	MDR	both lungs	4	multiple (4.7 x 2.8)	3	1	1	0	+	0	++
23	m	18	+				Recent	TB infiltrate	-	N/A	UL: S_{1+2}	1	none	0	0	0	++	N	N	N
24	f	53	+				Chronic	TB infiltrate	+	MDR	UL; UR	2	none	0	1	0	0	N	0	N
25	f	28	+				Recent	TB infiltrate	+	MDR	UR: S_1,S_2	1	none	0	0	0	+	N	N	N
26	f	30	+				Recent	Tuberculoma	-	ND	UR	2	none	0	0	0	+	N	N	N
27	m	33	+				Recent	TB infiltrate	+	s	UR: S_2	1	none	0	3	1	+	N	+	++
28	f	33	+				Recent	TB infiltrate	+	MDR	UL: S_{1+2}	1	one small	1	1	0	+	N	++	N
29	m	60	+				Recent	Tuberculoma	+	s	UL: S_3	1	none	0	0	0	+	N	N	N
30	f	43	+				Recent	TB infiltrate	+++	MDR	UL: S_{1+2},S_4,S_5; UR: S_2	2	multiple (3.5 x 2.0)	3	2	1	+	0	+	0
31	f	30	+				Recent	TB infiltrate	+++	s	UR; UL: S_4; LL: S_{10}	3	one small	1	0	1	+	0	N	++
32	m	25	+				Recent	Caseous pn.	+	s	left lung	4	multiple, system	3	3	3	0	0	+	+
33	f	33	+		+		Chronic	Cavitary TB	+	MDR	UR; UL	2	multiple, system	3	2	3	+	0	+	+
34	m	47	+		+		Chronic	Cavitary TB	++	XDR	both lungs	4	multiple, system (4.0 x 1.6)	3	3	1	+	0	0	0
35	m	23	+		+		Recent	Cavitary TB	+++	s	UR	2	multiple (3.0 x 2.5)	3	3	2	+	+	++	++
36	m	71	+		+		Recent	Caseous pn.	+++	MDR	both lungs	4	multiple, system (8.8 x 3.0)	3	3	3	0	0	+	+
37	m	33	+		+		Chronic	Cavitary TB	+++	XDR	both lungs	4	multiple, system	3	3	3	0	0	+	+
38	f	29	+		+		Chronic	Cavitary TB	++	XDR	both lungs	4	multiple, system	3	2	3	+	0	0	+
39	f	31	+		+		Recent	TB infiltrate	++	MDR	UL: S_{1+2}	1	multiple, small	2	2	2	+	+	++	++
40	m	62	+		+		Recent	Caseous pn.	++	MDR	UR; UL; MR; LL	4	multiple, system	3	2	3	+	0	0	+
41	f	39	+		+		Chronic	TB infiltrate	+	s	UL: S_{1+2}, S_4, S_5; LL: S_6	2	two small	2	0	0	+	+	N	N
42	f	35	+		+		Recent	TB infiltrate	++	s	left lung	4	multiple, system (1.7 x 0.9)	3	3	0	+	++	+	N
43	m	32	+		+		Recent	TB infiltrate	++	s	UR; UL	2	multiple (2.5 x 1.3)	3	1	2	+	+	++	++
44	f	24	+		+		Recent	TB infiltrate	++	s	UR: S_1,S_2; UL:S_{1+2}	1	multiple (3.5 x 3.5)	3	3	1	+	+	+	++
45	f	26	+		+		Recent	TB infiltrate	+	s	UR: $S_{1,}S_2$; MR: S_5	1	two small	2	1	1	+	++	0	++
46	f	30	+		+		Recent	TB infiltrate	+	MDR	UR: S_2; LR: S_8; UL: S_{1+2},S_4,S_5; LL: S_6	2	multiple, system (2.0 x 2.0)	3	2	1	0	0	0	0
47	f	44	+		+		Recent	TB infiltrate	++	MDR	left lung	4	three (2.9 x 1.6)	3	3	1	+	0	++	++
48	m	37	+		+		Chronic	Cavitary TB	++	MDR	both lungs	4	multiple (2.5 x 1.5)	3	1	1	++	0	0	++
49	m	21	+		+		Recent	TB infiltrate	+++	MDR	both lungs	4	two small	2	3	2	++	++	++	++
50	m	49	+		+		Recent	Caseous pn.	+	MDR	both lungs	4	multiple (2.1 x 1.4)	3	3	3	0	0	0	0
52	f	30		+	+		Recent	Caseous pn.	+++	s	left lung; UR	4	multiple (2.5 x 2.5)	3	3	3	+	0	++	++
54	f	31		+	+		Recent	TB infiltrate	+	DR	UR: S_1, S_2	1	multiple (2.0 x 2.0)	3	1	0	+	0	++	N
51	m	76		+	+		Chronic	TB infiltrate	+	MDR	UR: S_1; UL: S_{1+2}; LL: S_6	2	multiple (2.0 x 2.0)	3	2	1	0	0	++	++
53	f	33		+	+		Recent	TB infiltrate	+	DR	LR: S_{10}; LL: S_6, S_8	1	multiple (1.5 x 3.0)	3	3	1	++	++	0	++
55	f	25		+			Recent	Tuberculoma	+	MDR	UR: S_2	1	none	0	0	0	++	N	N	N
56	f	33		+			Chronic	Cavitary TB	+	s	UR; UL	2	two small	2	0	0	+	+	N	N
57	m	18		+			Recent	TB infiltrate	++	s	UL	2	one small	1	0	0	++	+	N	N
58	m	45		+			Recent	TB infiltrate	+	s	left lung	4	none	0	0	0	++	N	N	N
59	m	30		+			Recent	TB infiltrate	+	DR	UR: S_1; UL: S_{1+2}	1	multiple small	2	1	1	+	+	++	++
60	m	27		+			Recent	TB infiltrate	+	DR	UL: S_{1+2}	1	one small	1	1	1	+	+	++	0
61	m	29		+			Recent	TB infiltrate	+++	MDR	UR: $S_{1,}S_{2,}S_3$; MR: S_4; LR: S_6, S_{10}	2	one (3.0 x 4.0)	2	2	2	0	0	+	0
62	m	43		+			Recent	TB infiltrate	+++	MDR	UR; UL; MR	3	multiple (4.0 x 5.0)	3	3	2	0	0	+	+
63	m	34				+	Chronic	Cavitary TB	+++	s	both lungs	4	multiple,system (5.0 x 5.0)	3	3	3	+ (surgery)	+ (surgery)	++	++
64	m	21				+	Chronic	Cavitary TB	+	DR	left lung; UR	4	multiple, small	2	1	1	+ (surgery)	+ (surgery)	++	++
65	f	25				+	Recent	Cavitary TB	+	s	UL:S_{1+2},S_4,S_5; LL:S_6,S_{10}	2	multiple (3.0 x 2.3)	3	0	0	+ (surgery)	+ (surgery)	N	N
66	m	49				+	Recent	Tuberculoma	-	ND	UR: S_1,S_2,S_3	1	none	0	0	0	+ (surgery)	N	N	N
67	m	51				+	Chronic	Cavitary TB	+++	XDR	UR; UL	2	multiple,system (2.0 x 2.0)	3	3	2	+ (surgery)	+ (surgery)	+	++
68	f	38				+	Chronic	Cavitary TB	-	ND	MR; UR:S_3; LR:S_6; LL:S_8,S_9,S_{10}	2	multiple, small	2	0	1	+ (surgery)	+ (surgery)	N	++
69	f	33				+	Chronic	Cavitary TB	+	s	UR; UL:S_{1+2}	2	two small	2	0	0	+ (surgery)	+ (surgery)	N	N
70	f	28				+	Chronic	Tuberculoma	-	ND	UL:S_{1+2}; UR	2	none	0	1	0	+ (surgery)	N	++	N
