# Supplementary material for: Mtb-Specific CD27low CD4 T Cells as Markers of Lung Tissue Destruction during Pulmonary Tuberculosis in Humans
Source: PLoS One. 2012 Aug 24;7(8):e43733. doi: 10.1371/journal.pone.0043733 (PMC3427145; doi:10.1371/journal.pone.0043733)
Supplement: Table S2 — Characterization of TB contacts and Mtb -unexposed participants. 1Time of work in TB hospital (years). 2Not applicable. (PDF) [file pone.0043733.s002.pdf]

Table S2. Characterization of TB contacts and *Mtb*- unexposed participants.

TB contacts	Gender	Age	Duration of TB exposure[1]	Result of QFT test
**1**	f	29	1	-
**2**	m	42	4	-
**3**	f	61	35	+
**4**	m	34	2	-
**5**	f	63	38	-
**6**	f	74	51	+
**7**	f	52	33	+
**8**	f	35	11	-
**9**	f	28	7	+
**10**	f	60	15	-
**11**	f	40	12	+
**12**	f	43	6	-
**13**	f	70	1,5	+
**14**	f	54	1,5	+
**15**	f	45	7	+
**16**	f	73	1	-
**17**	f	41	6	-
**18**	m	35	12	+
**19**	m	48	20	+
**20**	f	25	3	-
**21**	m	40	15	-
**Mtb-unexposed**				
**1**	m	25	NA[2]	-
**2**	f	51	NA	-
**3**	f	30	NA	-
**4**	f	22	NA	-
**5**	m	23	NA	-
**6**	f	19	NA	-
**7**	m	21	NA	-
**8**	m	21	NA	-
**9**	f	58	NA	-
**10**	f	52	NA	-
**11**	m	23	NA	-
**12**	m	71	NA	-
**13**	m	28	NA	-
**14**	f	60	NA	-
**15**	f	61	NA	-
