# Supplementary material for: Mtb-Specific CD27low CD4 T Cells as Markers of Lung Tissue Destruction during Pulmonary Tuberculosis in Humans
Source: PLoS One. 2012 Aug 24;7(8):e43733. doi: 10.1371/journal.pone.0043733 (PMC3427145; doi:10.1371/journal.pone.0043733)
Supplement: Table S3 — Lack of correlation between TB manifestations and numbers of CD27low IFN-γ+ cells in the blood of TB patients. None of the analyzed factors correlated significantly with the numbers of CD27lowIFN-γ+ cells in the blood of TB patients. Initial analysis was performed in 50 patients. Subsequently, 12 patients from validation cohort were added (n = 62). In both cohorts, similar results were obtained (shown are results obtained in 50 patients). rho, Spearman coefficient, p, significance value of the test. (PDF) [file pone.0043733.s003.pdf]

**Table S3. Lack of correlation between TB manifestations and numbers of CD27$^{low}$ IFN-$\gamma^{+}$ cells in the blood of TB patients.**

| Factors | Simple correlation (Spearman) | |
|---|---|---|
| | **rho** | **p-value** |
| Lung tissue destruction | 0.23 | 0.11 |
| Clinical TB severity | 0.16 | 0.26 |
| Hematology abnormalities | 0.18 | 0.22 |
| Sputum *Mtb* positivity | -0.10 | 0.50 |
| TB extent | 0.16 | 0.28 |
| *Mtb* multi-drug resistance | -0.04 | 0.78 |
| TB duration | -0.11 | 0.45 |
