# Supplementary material for: Mtb-Specific CD27low CD4 T Cells as Markers of Lung Tissue Destruction during Pulmonary Tuberculosis in Humans
Source: PLoS One. 2012 Aug 24;7(8):e43733. doi: 10.1371/journal.pone.0043733 (PMC3427145; doi:10.1371/journal.pone.0043733)
Supplement: Table S4 — Percentages and numbers of IFN-γ+ and CD27low IFN-γ+ cells in the lungs and in blood of surgery operated patients. Blood and lung cells were analyzed on the day of lung surgery. Indicated are percentages and numbers (per 1 million of acquired cells) of IFN-γ+ and CD27lowIFN-γ+ cells. 1Fold differences between maximal and minimal values in the group. 2Correlations were analyzed between: the indicated cells in the lungs and blood; lung destruction and indicated cells; TB severity and indicated cells. (PDF) [file pone.0043733.s004.pdf]

Table S4. Percentages and numbers of IFN-γ$^+$ and CD27^{low} IFN-γ$^+$ cells in the lungs and in blood of surgery operated patients.

	IFN-γ$^+$ cells						CD27^{low} IFN-γ$^+$ cells					
**Patient/ parameter**	**Percentages (%)**			**Numbers**			**Percentages (%)**			**Numbers**		
	**Blood**	**Lung**	**LN**	**Blood**	**Lung**	**LN**	**Blood**	**Lung**	**LN**	**Blood**	**Lung**	**LN**
**Analysis of individual patients:**												
**Patient A**	0,37	10,6	0,14	472	3471	445	66,4	96,1	45.2	313	3335	201
**Patient B**	0,21	18,6	ND	139	20935	ND	53,8	83,3	ND	75	17439	ND
**Patient C**	0,64	6,98	ND	1536	23677	ND	70,5	97,6	ND	1083	23108	ND
**Patient D**	0,70	3,04	ND	894	842	ND	47,5	88,9	ND	425	748	ND
**Patient E**	1,10	1,65	ND	833	1623	ND	73,5	76,7	ND	612	1245	ND
**Patient F**	0,21	0,69	0,22	143	1034	728	45,5	94,1	45,2	65	973	329
**Patient G**	0,40	1,74	ND	603	3554	ND	48,4	83,3	ND	292	2960	ND
**Patient H**	0,21	5,65	ND	232	5221	ND	29,5	91,9	ND	68	4798	ND
**Statistical analysis:**												
**Median**	0,39	4,35	ND	538	3513	ND	51,1	90,4	ND	303	3148	ND
**Inter-patient differences[1]**	5,24	26,96	ND	11	28	ND	2,5	1,3	ND	17	31	ND
**Coefficient of variation**	66%	99%	ND	79%	123%	ND	27%	8%	ND	95%	125%	ND
**Correlations[2]:**												
**Lung *vs* blood, rho**	-0,27		ND	-0,07		ND	-0,11		ND	0,12		ND
**p-value**	0,52		ND	0,88		ND	0,80		ND	0,79		ND
**Destruction *vs* cells, rho**	0,35	0,11	ND	0,28	0,24	ND	**0,88**	0,19	ND	0,48	0,31	ND
**p-value**	0,40	0,79	ND	0,51	0,57	ND	**0,01**	0,65	ND	0,23	0,45	ND
**TB severity *vs* cells, rho**	-0,05	0,06	ND	-0,36	-0,24	ND	0,46	-0,09	ND	-0,17	-0,10	ND
**p-value**	0,90	0,88	ND	0,38	0,56	ND	0,25	0,83	ND	0,69	0,81	ND
